# Inpatient neurosurgical mortality in germany: a comprehensive analysis of 2023 in-hospital data

**DOI:** 10.1007/s10143-025-03664-1

**Published:** 2025-06-23

**Authors:** Marcel A. Kamp, Christine Jungk, Matthias Schneider, Georgia Fehler, Antonio Santacroce, N. Dinc, Florian H. Ebner, Christiane von Sass

**Affiliations:** 1 Brandenburg Medical School Theodor Fontane and Faculty of Health Sciences Brandenburg, Neuruppin, Germany; 2 Department of Palliative and Neuropalliative Care, Immanuel Clinic Rüdersdorf, University Hospital of the Brandenburg Medical School Theodor Fontane, Rüdersdorf bei Berlin, Seebad 82/83, 15562 Rüdersdorf near Berlin, Germany; 3 Department of Neurosurgery, University Hospital Heidelberg, Heidelberg, Germany; 4 Department of Neurosurgery, Medical Faculty, Heidelberg University, Heidelberg, Germany; 5 Department of Neurosurgery, University Hospital Bonn, Bonn, Germany; 6 Brandenburg Medical School Theodor Fontane, Neuruppin, Germany; 7 European Radiosurgery Center Munich, Munich, Germany; 8 Department of Neurosurgery, St. Barbara-Klinik Hamm-Heessen, Hamm, Germany; 9 Department of Medicine, Faculty of Health, Witten/Herdecke University, Witten, Germany; 10 Department of Neurosurgery, Jena University Hospital, Friedrich-Schiller-University, Jena, Germany; 11 Department of Neurosurgery, Alfried Krupp Hospital, Essen, Germany

**Keywords:** Mortality, Neurosurgery, Palliative care, Gender, Gender gap, Neurooncology, Subarachnoid hemorrhage, Case fatality, Germany

## Abstract

**Background:**

Neurosurgical conditions and procedures are associated with varying in-hospital mortality rates, which represent one of several quality indicators. This study aims to determine and report in-hospital mortality rates across German neurosurgical departments in 2023.

**Methods:**

A cross-sectional analysis of all neurosurgical cases treated in Germany in 2023 was conducted using nationwide hospital billing data reported under § 21 of the Hospital Remuneration Act. In-hospital mortality was defined as death during hospitalization (discharge status: deceased).

**Results:**

Neurosurgical departments treated 222,158 inpatient cases, with 49% female and 48% aged ≥ 65 years. The overall mortality rate was 3.8% (8,338 cases), with significantly lower rates in females (3.3% vs. 4.2%, *p* < 0.0001). The most common fatal diagnoses included traumatic subdural hematomas (1,278 cases), subcortical intracerebral hemorrhages (611 cases) and traumatic subarachnoid hemorrhages (504 cases). Mortality rates varied by diagnosis: malignant brain tumors (4%), cerebral metastases (6%), benign meningeal tumors (1.3%), non-traumatic subarachnoid hemorrhages (7%), intracerebral hemorrhages (29%), and traumatic subdural hematomas (12%). Mortality for selected procedures was 3% for primary brain tumor resections, 9% for vascular reconstructions, 1% for spinal fusions, 2% for dynamic stabilizations, and 4% for vertebral body replacements.

**Conclusions:**

This study analyzes and reports neurosurgical in-hospital mortality rates in Germany, providing a national benchmark that may inform clinicians, policymakers, and patients. While the use of administrative billing data imposes inherent limitations — particularly regarding clinical detail and causality — the findings may offer a foundation for future research. Subsequent studies should aim to explore disease- and procedure-specific mortality more granularly and may identify underlying risk factors.

**Clinical trial number:**

Not applicable.

## Introduction

Healthcare quality has gained significant importance in medicine and neurosurgery in recent years. Donabedian’s framework of structure, process, and outcome quality remains central to evaluating healthcare quality [1]. Structural quality refers to the organizational resources, human resources, and technical infrastructure that form the foundation of medical care. Process quality involves the procedures and actions taken during patient care, while outcome quality reflects the end results of care, providing a measurable indicator of the effectiveness of both the structure and processes involved [1].

Perioperative mortality has long been recognized as one of several key indicators of outcome quality in neurosurgical care and is used in studies, research, and quality assurance programs [2, 3, 4, 5, 6]. However, neurosurgery frequently involves conditions with inherently high mortality rates, such as subarachnoid hemorrhage, intracerebral hemorrhage, and traumatic brain injury. Mortality rates are influenced not only by the expertise of the surgical team and healthcare facility but also by patient-specific factors, including the underlying condition, age, comorbidities, and other relevant variables.

Despite the significance of mortality as a quality indicator, no nationwide data on in-hospital neurosurgical mortality in Germany have been published to date. This study aims to fill this gap by providing a comprehensive analysis of inpatient neurosurgical mortality in 2023 based on billing data, including calculations of mortality rates for common neurosurgical conditions and procedures.

## Methods

### Ethical approval and data accessibility

The study complied with the ethical standards set in the 1964 Declaration of Helsinki and its subsequent revisions. Approval for the study protocol was granted by the institutional and local ethics committee at Brandenburg Medical School, Germany (Study ID: 190032024-ANF). The findings align with the STROBE guidelines for observational studies [16].

### Study design, setting, and data sources

This cross-sectional study utilized aggregated data from all hospitalizations across Germany in 2023. Data were based on the § 21 of the Hospital Remuneration Act and sourced from the Institute for the Remuneration System in the Hospital Sector (InEK GmbH, Siegburg, Germany).

### Cohort definition, participants, and study size

The cohort comprised hospitalized cases that met the following criteria: (1) classified as inpatient neurosurgical cases, (2) treated during 2023, and (3) patients aged 18 years or older. Due to technical constraints, the analysis was limited to neurosurgical hospital cases, without access to individual patient-level data. The study size represents the total number of hospitalizations fulfilling these criteria in 2023. For comparison, the entire cohort of neurosurgical hospital cases in 2023 was analyzed alongside the subset of fatal cases.

### Definitions and variables

Neurosurgical departments were identified using their designated codes from Appendix 1 of the Federal Nursing Ordinance within the Federal Nursing Fee Ordinance dated December 31, 2003. These codes include 1700 (neurosurgery), 1790–1792 (neurosurgery without differentiation by subspecialty II – IV), and 3617 (intensive care medicine specializing in neurosurgery). Diagnoses were classified using the International Statistical Classification of Diseases and Related Health Problems, 10th Revision, German Modification (ICD-10-GM). In the German DRG system, each case is assigned a single primary diagnosis and may have multiple secondary diagnoses. Medical procedures and treatments were identified using their corresponding procedural codes (*Operationen- und Prozedurenschlüssel*,* OPS*) [17, 18]. The selection of analyzed comorbidities was guided by the Charlson Comorbidity Index [7].

The mortality rate was defined as the proportion of fatal hospital cases relative to the total number of cases within the corresponding cohort.

The analysis focused on the following variables:

- Total number of neurosurgical hospitalizations in 2023.
- Reasons for hospital discharge or transfer, documented according to Sect. 301, Paragraph 1, Sentence 1 of the German Social Code Book V.
- Total number of hospital cases within each cohort.
- Distribution of sex and age.
- Primary and secondary diagnoses, as well as medical procedures and treatments, for both all neurosurgical cases and fatal cases.
- Distribution of hospitals categorized by bed capacity and type of ownership.

### Addressing bias

Selection bias was minimized by including all DRG-based neurosurgical hospital cases in Germany for 2023. Patients outside the DRG system, such as foreign self-payers, are not included. Additionally, for data protection reasons, cases with an annual frequency of fewer than five were not available. However, given the large caseload of over 200,000 neurosurgical cases per year and the prevalence of the diagnoses and procedures analyzed, this limitation is unlikely to impact the findings significantly. To address measurement bias, neurosurgical cases were identified through their designated codes, along with associated diagnoses and treatments, using standardized ICD-10 and OPS coding systems. While billing data may introduce minor classification errors, the consistent application of coding standards mitigates this risk. Nonetheless, coding omissions, particularly for non-revenue-relevant DRG codes as secondary diagnoses and treatments, remain a possibility. This issue does not extend to primary diagnoses or the revenue-critical codes for surgical procedures. The study faced several limitations, including the lack of data on therapy timing, treatment sequencing, and patients’ overall and neurological health status. To account for disease severity, comorbidities were listed, and in-hospital deaths were analyzed alongside reasons for discharge, such as transfers to rehabilitation or nursing facilities.

### Data management and statistical analysis

Data were extracted from the InEK data browser and organized using Microsoft Excel for Mac (Version 16.93, Microsoft Corporation, Redmond, WA, USA). GraphPad Prism 9 for macOS (Version 9.5.1, GraphPad Software, La Jolla, CA, USA) was used for statistical analysis and visualization.

Descriptive statistics were used to calculate frequencies and ratios. The total number of cases for a given diagnosis included hospitalizations where the diagnosis appeared as either primary or secondary.

## Results

### Neurosurgical hospital cases

Neurosurgical hospitals in Germany managed 222,158 inpatient cases in 2023. Female patients accounted for 49.2% of these cases (109,191), while male patients comprised 50.8% (112,961). Three cases each were reported as diverse or of unknown gender. Patients aged 65 years or older constituted 48.2% of neurosurgical cases (106,993). The most common primary diagnoses included lumbar spinal canal stenosis, disc damage, and radiculopathies, which together accounted for 51,270 cases (23%). Additional common primary diagnoses included traumatic subdural hemorrhages (10,784 cases, 4.9%), cerebral aneurysms and subarachnoid hemorrhages (10,932 cases, 4.9%), cervical disc pathologies and radiculopathies (7,104 cases, 3.2%), benign neoplasms of the meninges (6,634 cases, 2.9%; D32.0), and secondary malignant neoplasms of the brain and meninges (5,944 cases, 2.7%). Comprehensive details of neurosurgical primary and secondary diagnoses are presented in Table 1 and supplementary Table 1.

**Table 1:** Overview over mortality rates of primary or secondary diagnosis

ICD-10-GM code		primary diagnoses all cases	primary diagnoses fatal cases	primary diagnoses fatal cases	primary diagnoses mortality		secondary diagnoses all cases	secondary diagnoses fatal cases	secondary diagnoses mortality
**Tumors**									
C71		Primary brain tumors	9800	385	3.9%		5601	242	4.3%
C79.3		Secondary tumors of brain and meninges	5944	344	5.8%		4886	433	8.9%
C79.5		Secondary malignant neoplasm of bone and bone marrow	2450	174	7.1%		3142	386	12.3%
C83.3		Diffuse large B-cell lymphoma	733	55	7.5%		532	46	8.6%
D32.0		Benign neoplasm: meninges	6634	85	1.3%		3858	66	1.7%
D33.3		Benign neoplasm: cranial nerves	1111	10	0.9%		792	6	0.8%
D35.2		Benign neoplasm: pituitary gland	2584	22	0.9%		1457	16	1.1%
**Neurovascular pathologies**									
I60		Subarachnoid hemorrhage	3844	660	17.2%		3083	635	20.6%
I61		Intracerebral hemorrhage	5513	1609	29.2%		6010	1692	28.2%
I62.00		Nontraumatic subdural hemorrhage: Acute	791	145	18.3%		1105	265	24.0%
I62.01		Nontraumatic subdural hemorrhage: Subacute	733	23	3.1%		493	32	6.5%
I62.02		Nontraumatic subdural hemorrhage: Chronic	3895	116	3.0%		2250	111	4.9%
I63		cerebral infarction	1574	199	12.6%		3856	868	22.5%
I67.10		Cerebral aneurysm (acquired)	7088	47	0.7%		4536	550	12.1%
I67.11		Cerebral arteriovenous fistula (acquired)	807	8	1.0%		450	16	3.6%
**Trauma**									
S06.0		Concussion	838	6	0.7%		2189	141	6.4%
S06.1		Traumatic brain edema	78	23	29.5%		1781	497	27.9%
S06.2		brain contusions and intraparenchymal hemorrhages	2386	378	15.8%		5448	1032	18.9%
S06.4		Epidural hemorrhage	771	26	3.4%		1256	127	10.1%
S06.5		Traumatic subdural hemorrhage	10784	1278	11.9%		8759	1195	13.6%
S06.6		Traumatic subarachnoid hemorrhage	5170	504	9.7%		5996	1016	16.9%
S06.70		Unconsciousness due to traumatic brain injury: Less than 30 minutes					2685	159	5.9%
S06.71		Unconsciousness due to traumatic brain injury: 30 minutes to 24 hours					332	96	28.9%
S06.72		Unconsciousness due to traumatic brain injury: More than 24 hours, with return to previous level of consciousness					103	18	17.5%
S06.73		Unconsciousness due to traumatic brain injury: More than 24 hours, without return to previous level of consciousness					548	344	62.8%
S06.79		Unconsciousness due to traumatic brain injury: Duration unspecified designated					3511	395	11.3%
S12		Fractures of the cervical spine	2620	214	8.2%		3290	370	11.2%
	S12.0	Fracture of the 1st cervical vertebra	306	27	8.8%		388	52	13.4%
	S12.1	Fracture of the 2nd cervical vertebra	1385	112	8.1%		933	106	11.4%
	S12.21	Fracture of the 3rd cervical vertebra	64	9	14.1%		181	27	14.9%
	S12.22	Fracture of the 4th cervical vertebra	124	16	12.9%		241	30	12.4%
	S12.23	Fracture of the 5th cervical vertebra	226	19	8.4%		426	52	12.2%
	S12.24	Fracture of the 6th cervical vertebra	307	24	7.8%		580	53	9.1%
	S12.25	Fracture of the 7th cervical vertebra	208	7	3.4%		520	50	9.6%
	S12.7	Multiple fractures of the cervical spine					21		0.0%
	S12.8	Fracture of other parts in the area of ​​the neck					20	6	30.0%
	S12.9	Fracture in the area of ​​the neck, part unspecified	6				39	9	23.1%
S13.0		Traumatic rupture of a cervical disc	89				316	30	9.5%
S13		Dislocation of cervical vertebrae	118	0			327	26	8.0%
S22		Fracture of the thoracic spine	1659	40	2.4%		2830	268	9.5%
S32		Fracture of the lumbar spine and the sacrum	2308	34	1.5%		3113	215	6.9%
**Infections**									
G06.0		Intracranial abscess and intracranial granuloma	1213	50	4.1%		1053	86	8.2%
G06.1		Intraspinal Abscess and intraspinal granuloma	709	37	5.2%		1108	96	8.7%
G06.2		Extradural and subdural abscess, unspecified	340	9	2.6%		419	32	7.6%
M46.2–5		Infections of the spine	2252	128	5.7%		2045	179	8.8%
A40 - A41		Sepsis	173	55	31.8%		2309	826	35.8%
R65.0		Systemic inflammatory response syndrome [SIRS] of infectious origin without organ complications					1010	118	11.7%
R65.1		Systemic inflammatory response syndrome [SIRS] of infectious origin with organ complications					2143	792	37.0%
R65.2		Systemic inflammatory response syndrome [SIRS] of non-infectious origin without organ complications					125	10	8.0%
R65.3		Systemic inflammatory response syndrome [SIRS] of non-infectious origin with organ complications					202	101	50.0%
**Degenerative cervical spine diseases**									
M42.12, M43.12,.22,.82,.92		Cervical sponylosis & osteochondrosis	1024		0.0%		2980	32	1.1%
M48.02		Spinal (canal) stenosis: cervical region	6017	36	0.6%		5541	103	1.9%
M50		Cervical disc damage	6104				4658	30	0.6%
M53.22		Spinal instability: cervical region	113				769	30	3.9%
**Degenerative lumbar spine diseases**									
M42.16-17, M47.15-16,. 26–27;.86–87, -96-96		lumbar and lumbosacral sponylosis & osteochondrosis	3400				6909	26	0.4%
M43.16,.17		Spondylolisthesis: lumbar & lumbosacral region	2261	0			4910	28	
M48.06		Spinal (canal) stenosis: lumbar region	22870	42	0.2%		16487	136	0.8%
M51.1		Lumbar and other disc damage with radiculopathy	20592	19	0.1%		10283	25	0.2%
M53.26		Spinal instability: lumbar region	623		0.0%		2203	17	0.8%
M54.16		Radiculopathy: lumbar region	918		0.0%		1472	11	0.7%
M54.17		Radiculopathy: lumbosacral region	1661		0.0%		2023		0.0%
**Hydrocephalus**									
G81.1		Hydrocephalus communicans	18		0.0%		2097	119	5.7%
G81.9		Hydrocephalus occlusus					2302	265	11.5%
G91.0		Other hydrocephalus	412	10	2.4%		1341	151	11.3%
G91.1		Hydrocephalus occlusus	720	28	3.9%		3163	625	19.8%
G91.8		Sonstiger Hydrozephalus	727	13	1.8%		2801	329	11.7%
G93.6		Cerebral edema	147	13	8.8%		12543	1622	12.9%
**Coagulation disorders**									
D68.32		Hemorrhagic diathesis due to increased antibodies against other coagulation factors					10		0.0%
D68.33		Hemorrhagic diathesis due to coumarins (vitamin K antagonists)					1005	209	20.8%
D68.34		Hemorrhagic diathesis due to heparins					145	36	24.8%
D68.35		Hemorrhagic diathesis due to other anticoagulants					2573	580	22.5%
D68.4		Acquired deficiency of coagulation factors					4179	984	23.5%
D68.8		Other specified coagulopathies					1020	261	25.6%
D69.58		Other secondary thrombocytopenias, not designated as transfusion refractory	9		0.0%		1716	457	26.6%
D69.80		Hemorrhagic diathesis due to platelet aggregation inhibitors					1483	243	16.4%
Neurological and clinical symptoms*									
G81		Hemiparesis and hemiplegia	36				17807	1957	
G82		Paraparesis and paraplegia, tetraparesis and tetraplegia	438	0			8520	581	
G83		Other paralysis syndromes	179	0			11609	468	
G90.2		Horner's syndrome					68		
R13.9		Other and unspecified dysphagia					4033	464	11.5%
R15		Fescal incontinence					6671	798	12.0%
R20.1		Hypoesthesia of the skin					11641	96	0.8%
R20.2		Skin paraesthesia					4192	27	0.6%
R26.3		Immobility					5251	551	10.5%
R26.8		Other and unspecified disorders of gait and mobility					11410	221	1.9%
R29.6		Tendency to fall, not elsewhere classified					3595	248	6.9%
R32		Unspecified urinary incontinence					7157	603	8.4%
R40.0		Somnolence					4847	1001	20.7%
R41.0		Disorientation, unspecified					5131	364	7.1%
R42		Dizziness and Dizziness					6186	121	2.0%
R47.0		Dysphasia and aphasia					11374	980	8.6%
R47.1		Dysarthria and anarthria					5368	537	10.0%
R51		Headache					10526	247	2.3%
R52.2		Other chronic pain					4255	103	2.4%
**Other**									
D62		Acute hemorrhagic anemia					10738	2053	19.1%
I20 - I25		Ischemic heart disease	92	7	7.6%		20689	1527	7.4%
	I21	Acute myocardial infarction	62	7	11.3%		534	132	24.7%
I26		Pulmonary embolism	27	0	0.0%		1571	289	18.4%
N17.91		Acute renal failure, unspecified: Stage 1					1507	338	22.4%
N17.92		Acute renal failure, unspecified: Stage 2	11		0.0%		1094	297	27.1%
N17.93		Acute renal failure, unspecified: Stage 3	30		0.0%		1289	571	44.3%
R18		Ascites	8		0.0%		690	230	33.3%
U07.1		COVID-19, virus detected					4587	445	9.7%

* The “Neurological and Clinical Symptoms” section presents relevant codes to indicate neurological impairment. Since these codes do partially not affect hospital reimbursement, they are likely to be inconsistently coded in clinical practice, suggesting probable underreporting of such symptoms

**Fig. 1:**
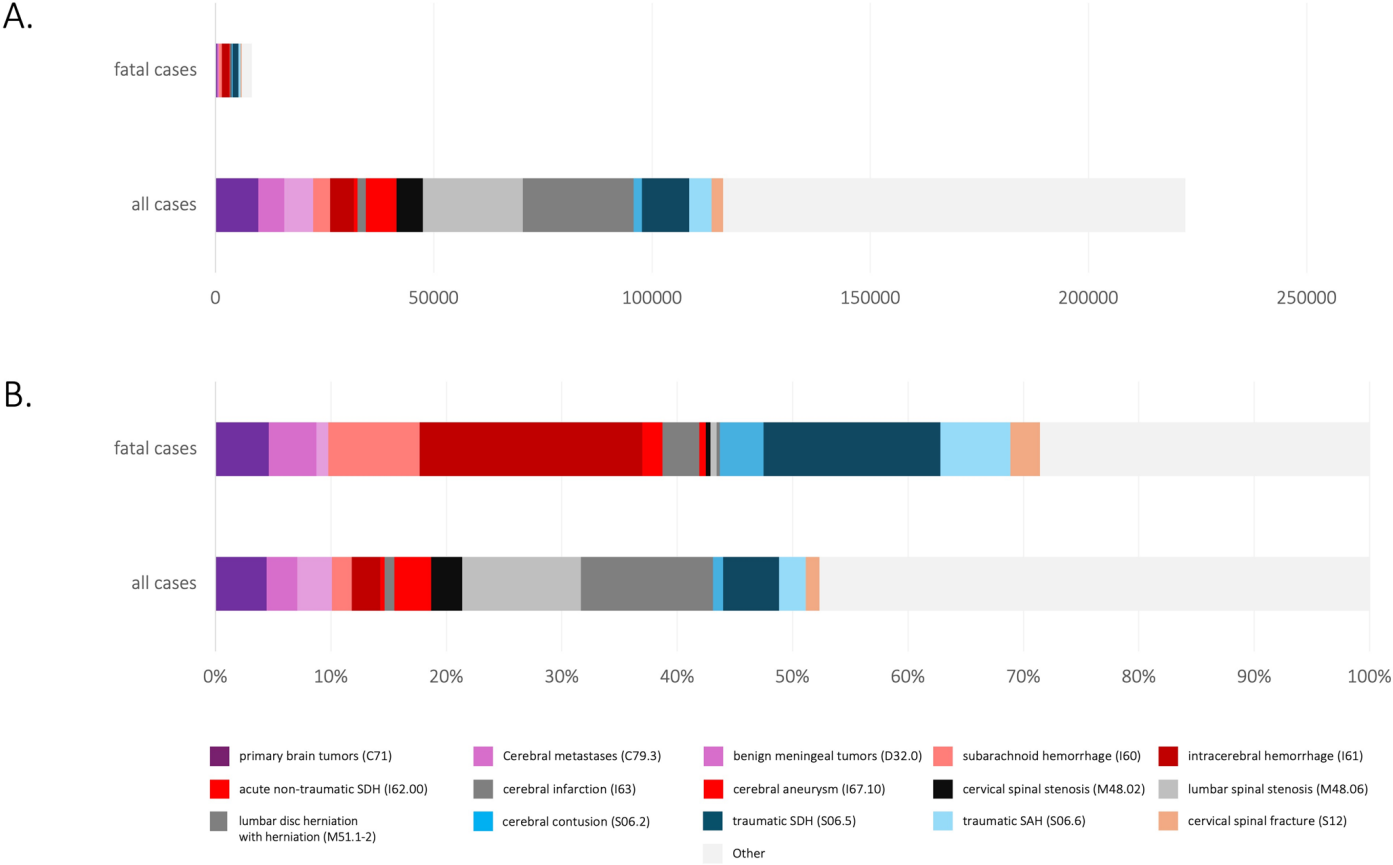
Tree diagram illustrating common primary diagnoses of all and fatal neurosurgical cases

The most frequent surgical procedures performed in neurosurgical inpatient settings were spinal surgeries (Fig. 1). These included the removal of intervertebral disc tissue in 46,992 cases, removal of bone tissue and joints during spinal surgery in 31,098 cases, and bony decompressions in 42,486 cases. Spondylodesis and dynamic stabilization procedures were performed in 14,300 and 42,486 cases, respectively, with multiple use of different secondary OPS codes for single procedures being common. Common cranial interventions included drainage of subdural hematomas (10,755 cases), operations for primary brain tumors (7,627 cases), secondary brain tumors (6,099 cases), and meningeal tumors (6,769 cases). Additionally, 5,801 cases involved closure and reconstruction of cerebral blood vessels.

### Hospital discharges

**Fig. 2:**
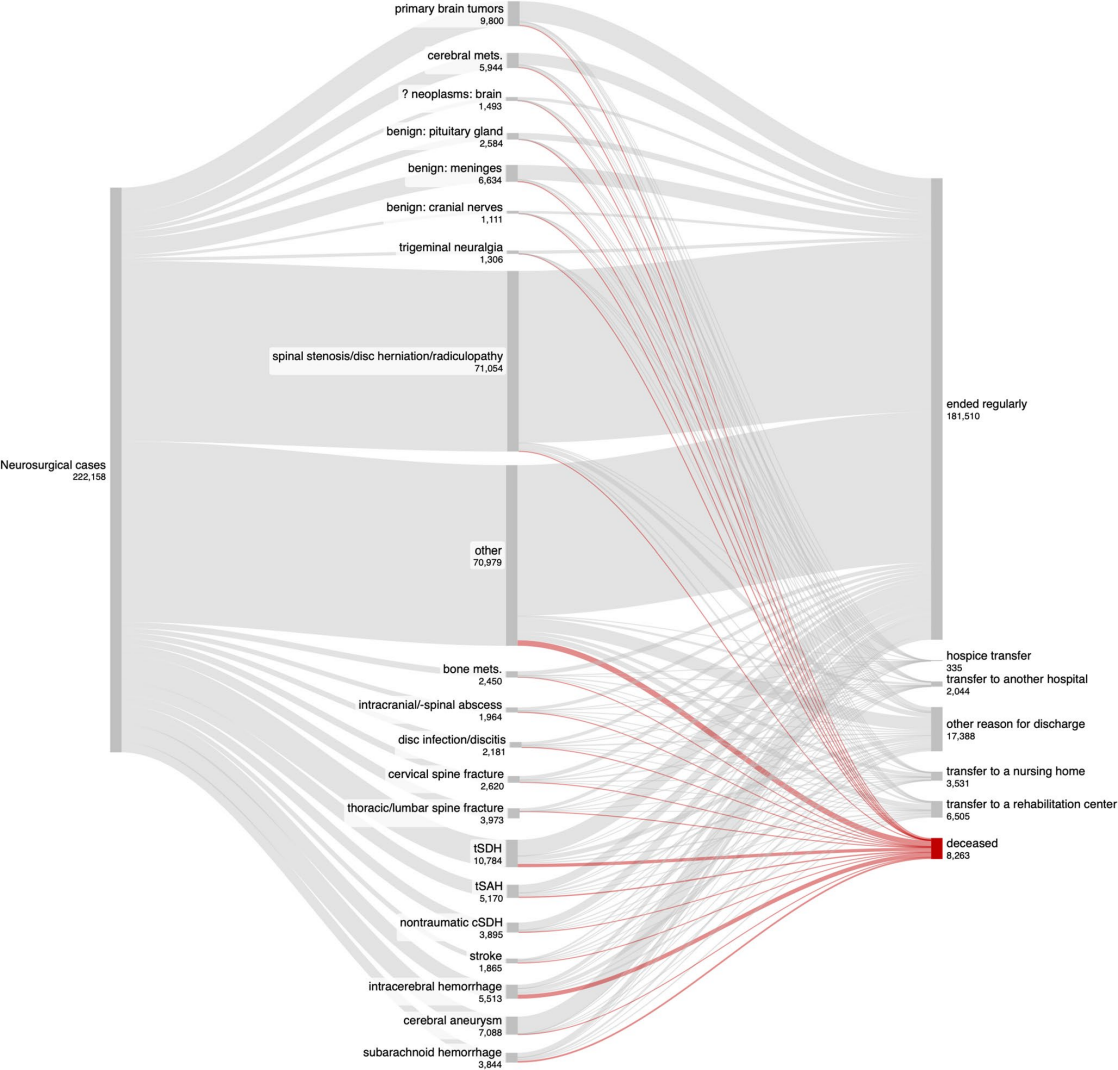
Sankey diagram illustrating common primary diagnoses and hospital discharge reasons of neurosurgical cases in Germany

In most cases, patients (82.5%; 183,269 cases) were discharged home after treatment. A minority of patients was transferred to rehabilitation facilities (3%; 6,613 cases), nursing homes (1.6%; 3,640 cases), or other hospitals (0.9%; 2,066 cases). Treatment was terminated against medical advice in 0.9% of cases (2,029, Table 2; Fig. 2). Other discharge reasons accounted for 16,203 cases (8.8%).

**Table 2:** Overview over hospital discharges of neurosurgical cases and related primary diagnoses

ICD-10-GM code	primary diagnoses	All cases	discharged home	transfer to another hospital	deceased	hospice transfer	rehabilitation facility	transfer to a nursing home
**All neurosurgical hospital cases**		222158	183269	2066	8338	335	6613	3640
**Primary diagnoses**								
C71	Malignant primary brain tumors	9800	8003	53	385	122	136	200
C79.3	Cerebral metastases	5944	4691	64	344	42	41	94
C79.5	Bone metastases	2450	1665	19	174	20	45	55
D32.0	Benign neoplasm: meninges	6634	5786	37	85		297	55
D33.3	Benign neoplasm: cranial nerves	1111	1024		10		36	
D35.2	Benign neoplasm: pituitary gland	2584	2450	19	22		27	7
D43.0-1	Neoplasm of uncertain or unknown behavior: brain, supratentorial	1493	1190	71	51	5	23	27
G06.0-1	Intracranial/-spinal abscess and granuloma	1922	1238	20	87	0	162	40
G50.0	Trigeminal neuralgia	1254	1228	6				6
I60	SAB	3844	1817	32	660	0	469	43
I61	ICH	5513	1460	18	1609	8	705	135
I62.02	Nontraumatic subdural hemorrhage: chronic	3895	2999	16	116		156	136
I63	Cerebral infarction	1865	655	0	266	0	333	61
I67.10	Cerebral aneurysm (acquired)	7088	6716	50	47		112	15
	Disc infection/discitis	2181	1273	11	128	0	141	46
	stenosis/disc damage/radiculopathy	71054	67648	461	113	0	737	221
S06.5	Traumatic subdural hemorrhage	10784	6359	152	1278	12	572	613
S06.6	Traumatic subarachnoid hemorrhage	5170	3208	121	504		271	262
S12	Cervical spine fracture	2620	1596	16	214	0	109	120
S22/32	Thoracic/lumbar spine fracture	3973	3132	28	74	0	108	108

### Mortality

A total of 8,338 patients (3.8% of all neurosurgical hospitalizations) died during inpatient treatment. Among these, 43% were female, and 75% were aged 65 years or older. The overall mortality rate was significantly lower for female patients (3.3%) compared to male patients (4.2%, *p* < 0.0001, Chi-square test).

The most common primary diagnoses in fatal cases included intracerebral hemorrhage (1,609 cases), traumatic subdural hematomas (1,278 cases), non-traumatic subarachnoid hemorrhages (660 cases), and traumatic subarachnoid hemorrhages (504 cases). Fatalities associated with primary brain tumors accounted for 384 cases (3.9%), while cerebral metastases contributed to 344 deaths.

Mortality rates depended on both the underlying pathology and whether it was coded as a primary or secondary diagnosis. Table 1 provides a detailed overview. Among diagnoses with ≥ 100 cases in 2023, intracerebral hemorrhage recorded the highest mortality rate as a primary diagnosis at 29.2%. Other neurovascular diseases also demonstrated elevated mortality rates. Non-traumatic subarachnoid hemorrhages showed a mortality rate of 17.2% when coded as the primary diagnosis, rising to 20.6% when coded as secondary. Similarly, cerebral infarctions had a primary diagnosis mortality rate of 12.6%, which increased to 22.5% for secondary diagnoses.

In the tumor group, primary brain tumors exhibited a mortality rate of 3.9% as a primary diagnosis and 4.3% as a secondary diagnosis. Secondary brain and meningeal tumors showed mortality rates of 5.8% for primary diagnoses and 8.9% for secondary diagnoses. Benign neoplasms, such as meningeal tumors, had significantly lower mortality rates at 1.3% and 1.7% for primary and secondary diagnoses, respectively.

Trauma-related pathologies demonstrated high mortality rates. Traumatic subdural hemorrhage had a primary diagnosis mortality rate of 11.9%, which rose to 13.9% for secondary diagnoses. Intraparenchymal hemorrhages and contusions showed mortality rates ranging from 15 to 20%, with 15.8% for primary diagnoses and 18.9% for secondary diagnoses. Spinal injuries exhibited mortality rates of 8.2% as primary diagnoses and 11.2% as secondary diagnoses. Cerebral and spinal infections displayed mortality rates ranging from 4 to 9%. Sepsis was associated with a high mortality rate with 31.8% when recorded as the primary diagnosis and 35.8% when coded as a secondary diagnosis.

Degenerative diseases, including lumbar and cervical spine disorders, had the lowest mortality rates. Lumbar degenerations showed mortality rates below 0.8% when coded as secondary diagnoses and 0.2% as primary diagnoses. Cervical degenerative pathologies exhibited slightly higher mortality rates, peaking at 1.9% for spinal canal stenosis and 3.9% for spinal instabilities, both coded as secondary diagnoses, and 0.6% for spinal canal stenosis coded as primary diagnoses.

Comorbidities were common among neurosurgical patients. Secondary diagnoses indicated diabetes mellitus in 14.5% of cases, renal failure or chronic kidney disease in 7%, and heart failure in 4.8%. Mortality rates varied substantially across comorbidity groups: patients with a secondary diagnosis of myocardial infarction had a mortality rate of 24.7%, while those with heart failure or renal failure/chronic kidney disease had rates slightly above 15%. Patients with gastrointestinal ulcers had a mortality rate of 14.5%. A detailed list of relevant comorbidities, categorized according to the Charlson Comorbidity Index, is provided in Table 3.

**Table 3:** List of relevant comorbidities, categorized according to the Charlson Comorbidity Index

ICD-10-GM code	Code description	primary diagnoses all cases	primary diagnoses fatal cases	primary diagnoses mortality
**Incision of the brain and meninges**				
5–013.0	Incision of the brain and meninges: Drainage of subdural fluid	2027	159	7.8%
5-013.1	Incision of the brain and meninges: Drainage of a subdural hematoma	10755	1029	9.6%
5-013.40	Incision of the brain and meninges: Drainage of an intracerebral hematoma: Open surgical	3285	772	23.5%
**Excision and destruction of diseased intracranial tissue**				
5–015.0	Excision and destruction of diseased intracranial tissue: Intracerebral tumor tissue, brain-specific	7627	216	2.8%
5-015.1	Excision and destruction of diseased intracranial tissue: Intracerebral tumor tissue, non-brain-specific	6099	310	5.1%
5-015.3-5	Exzision und Destruktion von erkranktem intrakraniellem Gewebe: Hirnhäute	6769	125	1.8%
**Cerebrospinal shunt**				
5-023.10	Cerebrospinal fluid shunt [shunt implantation]: Drainage into the peritoneal space: Ventriculoperitoneal	4919	145	2.9%
**Incision, excision, destruction and closure and reconstruction of intracranial blood vessels**				
5–025	Incision, excision, destruction and closure of intracranial blood vessels	3447	264	7.7%
5–026	Reconstruction of intracranial blood vessels	2354	211	9.0%
**Excision and destruction of diseased tissue of the spinal cord and spinal meninges**				
5–035	Excision and destruction of diseased tissue of the spinal cord and meninges	5737	175	3.1%
**Excision and resection of diseased pituitary tissue**				
5–075	Excision and resection of diseased tissue of the pituitary gland	2749	19	1.9%
**Spinal surgeries**				
5-831	Excision of diseased Intervertebral disc tissue	46992	231	0.5%
5-832	Excision of (diseased) bone and joint tissue of the spine	31098	339	1.1%
5-836	Spondylodesis	14300	206	1.4%
5-837	Vertebral body replacement	2071	81	3.9%
5-839.6	Other operations on the spine: Bony decompression of the spinal canal	42486	421	1.0%
5-83b	Osteosynthesis (dynamic stabilization) on the spine	39567	793	2.0%

### Procedures

Table 3 provides information on mortalities rates of neurosurgical procedures. Hospital cases involving tumor surgeries had mortality rates ranging between from under 2% (benign tumors of the meninges and pituitary gland) up to 5.1% for secondary brain tumors. Hematoma surgeries showed higher mortality rates, with 9.6% for subdural hematoma drainage and 23.5% for open intracerebral hematoma evacuation. Intracranial blood vessel reconstruction had a 9% mortality rate. Spinal surgeries had comparatively lower mortality rates, including 0.5% for disc surgery, 1.4% for spondylodesis, 2% for dynamic stabilization, and 3.9% for vertebral body replacement.

## Discussion

Our analysis of German neurosurgical hospital cases in 2023 reveals the following key findings:

1. Neurosurgical departments managed 1.5% of all adult hospital cases in Germany. The most frequent conditions treated included lumbar spinal canal stenosis and intervertebral disc pathologies, accounting for nearly 25% of all neurosurgical cases.
2. Among all cases, 3.8% of patients died during their hospital stay. A significant majority (83%) were discharged home, while 3% were transferred to rehabilitation facilities, and 1.6% to nursing homes.
3. Women demonstrated a significantly lower mortality rate than men (3.3% vs. 4.2%). Mortality rates also varied depending on the underlying pathology and whether the diagnosis was coded as primary or secondary. Intracerebral hemorrhages, subarachnoid hemorrhages, traumatic subdural hematomas, and cerebral contusions exhibited particularly high mortality rates.

In 2023, Germany recorded 8,338 neurosurgical fatalities out of a total of 222,158 neurosurgical cases, resulting in a mortality rate of 3.8%. This statistic encompasses all neurosurgical cases, including those managed conservatively and those identified as fatal upon admission. However, cases that included consultations without subsequent transfer to a neurosurgical clinic were not included in these data. Mortality was influenced by the patient’s sex, underlying pathology, procedures performed, and the coding of diagnoses as primary or secondary.

Numerous studies have examined neurosurgical mortality rates, reporting values that are generally comparable but vary considerably depending patient populations, procedural scope, and study design. Large-scale analyses from the American College of Surgeons National Surgical Quality Improvement Program (ACS-NSQIP) have documented 30-day mortality rates of 3.8% following cranial surgery and 1.6% for elective craniotomies [6, 8]. In England, data from 24 neurosurgical centers indicated 30-day mortality rates of 0.5% and 0.1% for elective cranial and spinal procedures, respectively [3]. In contrast, unplanned major surgeries showed significantly higher mortality, reaching up to 6.6% for cranial interventions [3]. The differences within these cohorts and our own are primarily attributable to variations in case mix, particularly the proportion of high-risk procedures and the presence of comorbidities. Furthermore, cited studies are partially limited to elective [3, 8] or cranial procedures [6, 8], whereas our cohort includes both elective and emergency surgeries, encompassing the full spectrum of neurosurgical interventions. In addition, while the cited studies primarily report 30-day mortality, our analysis is limited to in-hospital mortality, which may lead to differences in reported rates due to varying observation periods. Institutional studies from high-volume academic centers, such as Duke University Medical Center and Helsinki University Hospital, have reported in-hospital mortality rates ranging from 2.4 to 4.2%, with reductions observed following the implementation of targeted quality improvement programs [4, 5]. In German neurosurgical centers, overall in-hospital mortality rates have been reported at approximately 3.1–3.4%, with procedure-specific mortality rates as low as 0.4% [9, 10]. Data from Vienna, based on more than one thousand index neurosurgical procedures, reported mortality rates of 0.9% at 7 days, 2.5% at 30 days, and 4.7% at 90 days postoperatively [11].

In our cohort, high in-hospital mortality rates were observed for conditions such as intracerebral hemorrhage, subarachnoid hemorrhage, and traumatic brain injuries. These findings are consistent with previously published data, although exact rates differ across studies. For example, reported 30-day mortality for intracerebral hemorrhage ranges between 24% and 34% in European and U.S. registry studies [12, 13]. Subarachnoid hemorrhage mortality varies more widely, with hospital rates ranging from below 10% to over 30%, depending on study design, case severity, and coding practices [14, 15, 16, 17]. Variations in reported mortality rates can largely be attributed to differences in patient populations, treatment strategies, and definitions of mortality across studies. In Germany, in-hospital mortality for subarachnoid hemorrhage was 17.2% for primary diagnoses and 20.6% for secondary diagnoses. The higher in-hospital mortality was recorded when subarachnoid hemorrhage was coded as a secondary diagnosis, likely reflecting severe complications such as cerebral infarction. Tumor-related mortality in our data was considerably lower. Neurosurgical interventions for primary brain tumors showed approximately 4% mortality, while rates for cerebral metastases ranged from 5.8 to 8.9%, and for meningeal tumors from 1.3 to 1.7% in our present study. In the U.S., surgical brain tumor cases showed approximately 2% mortality across various series [24, 25, 26]. Cerebral metastases resections had an overall in-hospital mortality rate of 3.1% in the U.S. between 1988 and 2000 [27].

Sepsis was associated with disproportionately high mortality in our cohort. When recorded as the primary diagnosis, in-hospital mortality reached 31%, increasing to nearly 36% when documented as a secondary diagnosis. These figures exceed those reported in previous multicenter studies, where postoperative sepsis in neurosurgical patients was associated with mortality rates ranging from 7.7% to over 30%, depending on population characteristics and healthcare settings [18, 19, 20, 21]. In a large analysis of the ACS NSQIP database (2012–2015), patients with septic shock exhibited a mortality rate of 33% [18]. The reported incidence of sepsis also varied considerably, from less than 1% in our study to over 30% in a Chinese cohort. Beyond differences in patient populations, comorbidity profiles, and treatment strategies, discrepancies in sepsis definitions, the distinction between sepsis and septic shock in some studies, and the absence of a clinical definition in our analysis — relying solely on administrative billing codes — likely contribute to the observed variation in mortality rates.

Mortality associated with neurosurgical procedures serves as a significant quality indicator for evaluating neurosurgical treatments. It has been used as a benchmark in audits, studies, and quality assurance programs [2, 3, 4, 5, 6, 22]. However, outcome quality—encompassing mortality—represents only one aspect of healthcare evaluation. Donabedian’s framework highlights the equally important roles of structural and process quality in assessing treatment standards. Furthermore, relying solely on mortality as a quality metric has inherent limitations.

One major challenge lies in inconsistent definitions. While inpatient mortality is commonly reported, 30-day mortality is frequently used as an endpoint in studies [3, 4, 6]. Additionally, there have been calls to include 48-hour and 90-day mortality rates. The 48-hour metric more accurately captures surgical causes of death, whereas the 90-day endpoint offers a more comprehensive picture of cumulative outcomes [23]. Mortality rates also depend on numerous factors, such as the severity of the condition, patient age, comorbidities, and the clinical indication for surgery. Furthermore, it remains debatable whether neurosurgeons performing high-risk procedures — e.g. unavailable at other institutions —can and should be evaluated using mortality rates alone [23].

In the present study, male patients exhibited a significantly higher in-hospital mortality rate than female patients. However, based on the available administrative data, it is not possible to determine whether this reflects an intrinsically higher neurosurgical mortality risk among men or a greater prevalence of high-risk conditions within the male subgroup. The literature on gender-related differences in neurosurgical outcomes remains inconsistent. Several studies have reported no significant gender-based differences in mortality or complication rates across various neurosurgical populations, including those undergoing elective craniotomy, treatment for aneurysmal subarachnoid hemorrhage, clipping of unruptured aneurysms, and management of malignant gliomas (Brandi et al., 2022; Drexler et al., 2024; Goldberg et al., 2024; De Marchis et al., 2017). In contrast, a systematic review and meta-analysis in elective spine surgery found that male patients had a higher incidence of mortality and medical complications (Kumar et al., 2023). Moreover, a broader analysis of all adult hospitalizations in Germany revealed a higher mortality among men: while men accounted for 47.5% of total adult hospital cases (7,222,035 of 15,200,893), they represented 53.9% of all in-hospital deaths (236,915 of 439,315; Chi-square test, *p* < 0.0001). Future research should explore sex-specific factors influencing neurosurgical outcomes and also examine whether physician gender plays a role in patient prognosis.

Incorporating the patient perspective into quality assessment is essential. It remains unclear whether mortality rates adequately reflect patient priorities, as no studies have comprehensively addressed this issue. Meanwhile, patient-reported outcome measures (PROMs) have gained prominence in neurosurgery and its subspecialties [24, 25, 26, 27, 28, 29, 30]. PROMs allow for the assessment of symptoms, challenges, and stressors that matter to patients but might otherwise go unnoticed. These measures provide direct insights into health outcomes from the patient’s perspective. When combined with traditional indicators such as mortality rates, PROMs offer a more holistic understanding of healthcare quality and patient well-being.

This study establishes a benchmark for neurosurgical mortality in Germany and offers an overview of national trends. However, it permits only limited conclusions regarding factors influencing mortality. Detailed analysis of these factors by specific diagnoses or procedures is constrained by the nature of billing data and lies beyond the scope of this paper. Such analyses will be addressed in a subsequent study. Future efforts should aim to refine benchmarks and integrate patient-centered outcomes to enhance quality assessment in neurosurgical care. Emerging approaches using artificial intelligence (AI), including machine learning (ML) and artificial neural networks (ANNs), offer promising avenues for deeper insight. AI enables rapid processing of complex datasets, facilitating the detection of subtle patterns essential for risk stratification and clinical decision-making [31]. ANNs have already been successfully applied to predict outcomes in epilepsy, brain metastases, and lumbar spinal stenosis [32]. Moreover, ML algorithms have outperformed clinical experts in 58% of outcome measures, demonstrating their growing potential in neurosurgical research and practice [33, 34].

### Limitations

This study has several limitations:

1. The entire number of neurosurgical hospital cases do not equate to individual patients, as some may experience multiple hospitalizations within a year with varying treatments. However, cases involving deceased patients represent a unique case in this regard, as individuals generally experience only one instance of death, aligning the number of hospital cases with the number of patients in this specific scenario.
2. Hospital mortality was calculated without specifying causes of death. However, primary diagnoses likely reflect key factors influencing hospitalization outcomes.
3. Billing codes in this dataset are recorded shortly after discharge and linked to the overall hospital stay, without reference to specific time points. Consequently, we cannot reconstruct the sequence of diagnoses, procedures, or complications, nor determine how many revision surgeries were performed. To capture comorbidities, we applied the Charlson Comorbidity Index, which nonetheless precludes precise risk adjustment.
4. We have added codes for neurological and clinical symptoms to Table 1 to illustrate neurological and clinical impairments. As these codes partially do not influence hospital reimbursement, they are likely inconsistently coded in clinical practice, indicating probable underreporting of such symptoms.
5. In this study, we descriptively reported mortality rates for specific diagnoses and procedures based on billing codes within the entire neurosurgical cohort and the subset of fatal cases. We have not assessed correlations or causal relationships between individual conditions, procedures, and mortality. Future studies will aim to explore associations between comorbidities, age, gender, and specific diagnoses or procedures. However, such analyses will remain correlational and cannot determine causality.
6. The ICD-10-GM classification lacks a specific code for polytrauma, and the existing codes for multiple injuries do not allow for reliable identification or analysis of such cases. As a result, polytrauma could not be accurately captured in our dataset, which constitutes a limitation of this study.
7. This study reports only inpatient mortality rates due to the structure of the billing data, while definitions of mortality may vary depending on context. As the data cannot be validated against clinical records or linked to post-discharge outcomes, late mortality and long-term functional outcomes could not be assessed.
8. All operative and non-operative neurosurgical hospital cases were included. Mortality rates for specific diagnoses cannot therefore necessarily be associated with specific procedures and vice versa.
9. Data on outpatient care for neurosurgical patients are unavailable.
10. Findings depend on consistent coding in the InEK database, and misclassifications could introduce bias. Previous research, however, suggests high coding reliability in Germany [31]. Quality-of-life data are also lacking.

## Conclusion

This study provides the first nationwide analysis of in-hospital neurosurgical mortality rates in Germany, offering a benchmark for clinicians, policymakers, and patients. Neurosurgical cases accounted for 1.5% of adult inpatient admissions, with an overall mortality rate of 3.8% with higher rates in men than women and substantial variation across conditions — particularly higher rates in intracerebral hemorrhage and traumatic brain injury. While mortality is only one quality indicator among others, such as functional outcomes or patient-reported measures, it remains a relevant metric. Due to the use of administrative billing data, clinical detail and causal inference are limited. Nevertheless, the findings offer a valuable reference point and may guide future, more granular investigations into disease- and procedure-specific mortality and associated risk factors.

## Data Availability

No datasets were generated or analysed during the current study.

## Declarations

### Generative AI and AI-assisted technologies in the writing process

AI-assisted technology was neither used for the generation, evaluation or interpretation of the data presented in the manuscript, nor for the creation of text, figures or tables. AI-based tools (chat GPT) may have been used to improve language and text readability.
